# Medicine

**Published:** 1908-06

**Authors:** G. Parker


### Progress of the Medical Sciences.

### MEDICINE.

**Carbon Monoxide Poisoning.**—It has been evident for some time past that the importance of this substance as a lethal agent is increasing, and that the variety and complexity of the symptoms have been underrated. It is now produced in gigantic quantities in various processes, for not only is it a common cause of death in colliery explosions and from accidents at blast furnaces, but the recent introduction of electric furnaces, water gas, nickel carbonyl, and other factories has brought it everywhere among us. In the works of one company only, who get power from the

Niagara Falls, sixteen tons daily are evolved. In France the charcoal stove has long been a favourite method of suicide, but in England, and still more in America, the use of carburetted water gas as a means of enriching coal gas at a cheap rate has led to an immense increase of fatalities which are frequently unintentional. Nearly 15 per cent. of all the English gas works, private and municipal, together supply their gas mixed with this water gas in quantities varying from 3 to 60 per cent. in the different companies, and the total output exceeds 18,000,000 cubic feet. Now the danger of this is shown by the experience of America. In eighty-eight cities, the total population of which was equal to that of London, the deaths from pure coal gas only numbered three, but in Boston, Brooklyn, Chicago and San Francisco, where the proportion of water gas was very high, the deaths amounted to 935 in a smaller population. The gas quickly loses its odour, and easily passes through the subsoil or brick walls, so that the gas from a broken main may cause poisonous results in a house adjoining. In one instance the illness produced was thought to be typhoid (Pettenkofer), and the nurse and another patient were believed to have been infected by the first case. In fact, many instances of "sewer gas" and "blood poisoning" are probably due in reality to carbon monoxide, which has gained entrance through a defective pipe. Even pure coal gas contains a proportion of some 6 to 9 per cent., but water gas has often 50 per cent. The symptoms of poisoning come on insidiously, even in severe cases. There may be at first slight dyspnœa and palpitation, loss of perception of time, and of the power of recognising friends before the patient realises that anything is amiss. He may be excited, or simply drowsy, and then unconscious. Death may follow suddenly after muscular exertions in attempts to escape. The effects depend much on the amount of oxygen present. If this is low, carbonic oxide may cause death in a few seconds with convulsions, whereas in an atmosphere of oxygen Haldane and Mosso have shown that enormous amounts are tolerated, because the red cells are then less important, and much oxygen may be conveyed by the plasma.

A theoretical discussion has arisen as to whether CO has a directly poisonous action on the tissues, or merely shuts off all oxygen from them. The peculiarly stable compound it forms with hæmoglobin is well known, and the changes in the nervous system are numerous and important. Hence, besides immediate symptoms we see sometimes in those who recover mental and nerve affections lasting for weeks or years. Mott[1] has found most extensive cerebral hemorrhages and softenings, and fatty degeneration of other organs. Edsall and others describe corresponding symptoms—hemiplegia, dementia, hallucinations, altered reflexes, peripheral neuritis, and sudden heart failure. Permanent effects

[1] *Arch. Neurol.*, 1907, iii. 246.

may not be preceded by acute poisoning, as in a case described by Lewin.[1] Here a woman exposed to water gas suffered merely from vomiting and headache without any physical signs of illness, but remained mentally affected for many months. Scott[2] reported other instances where mania, melancholia and dementia followed acute poisoning. Gilman Thompson,[3] in a study of ninety cases, remarks on the general presence of pyrexia, preceded or followed by subnormal temperature, and of leucocytosis, which in fatal cases may reach 50,000. Coma may last for days, even in hopeful cases ; albuminuria, or glycosuria, and pneumonia are not infrequent.

Glaister[4] discusses the results of chronic poisoning, where, for instance, persons have worked under burners consuming much carburetted water gas, and thinks that to this is due the anæmia, anorexia, loss of nutrition, and mental apathy which so many of these operatives suffer from. He argues that where mixed or impure gas is supplied, an inspection of all pipes and fittings should be enforced. More injury to health is done, he thinks, from impure coal gas than from sewer gas. The question, though by no means settled, is important. If the benefits of fresh air are great, what are the active elements of impure air ? Are its effects chiefly due to carbon monoxide ? Louis Parkes[5] points out that carbonic acid has of itself little importance. Nature never allows us to take a breath free from it, and the high percentage in the pulmonary alveoli is always kept constant. The diminution of oxygen in crowded rooms never reaches that found in healthy places at high altitudes. No volatile organic poison has been discovered in expired air, and apart from the germs of phthisis and a few other diseases, no pathogenic organisms have been found. We have, therefore, either to invoke some unknown constituent, or to fall back on carbon monoxide. It is clear that in severe cases of poisoning by the latter, the lesions in the vascular and nervous systems are most extensive and lasting. Are the effects of small repeated doses a common cause of ill-health, though no pathological changes can be found ?

**Calmette's ophthalmic reaction** for the diagnosis of tuberculosis is of importance in various ways. In the first place, it may prove to be a great assistance in the early discovery of disease while easily curable, in which case its value will be incalculable. Again, just as with the Widal reaction, the pathological process may throw light on the physiology of immunity, and, finally, there are already indications that similar reactions will facilitate the diagnosis of other diseases. Thus Chantemesse has obtained a

[1] *Berl. klin. Wchnschr.*, 1907, xliv. 1367.
[2] *Lancet*, 1896, i. 217. [3] *Med. Rec.*, July, Sept., 1905 (?).
[4] *Lancet*, 1906, ii. 1578. [5] *Practitioner*, 1904, lxxiii. 550.

specific reaction in the eyes of typhoid patients by instillation of a typhoid antitoxin.

Widespread controversy has arisen as to the value and dangers of the test, but it has already been extensively used. Calmette, in January last, could point to 10,000 cases where it had been employed, and a steady flow of reports still continues. The idea was developed from some investigations of von Pirquet upon small-pox.[1] He noticed that the size of the papules produced by inoculating calf lymph varied with the concentration of the lymph used, and it occurred to him that similar results ought to follow the inoculation of the virus of other disease in susceptible people. He found that 25 per cent. of tuberculin did actually produce a similar papule, but only in tuberculous persons. It next appeared that the reaction was sufficiently regular to serve as a means of distinguishing tuberculous children, though with adults it was uncertain from the frequency of healed tuberculosis. From the results of one hundred *post-mortems* on children, he was able to assert that a positive result is certain proof of the existence of the disease, and a negative one strong evidence of its absence.

Calmette[2] then devised his plan of dropping into the conjunctival sac a one per cent. suspension in distilled water of tuberculin which had been precipitated by alcohol. He found that in tuberculous patients some redness and swelling resulted in a few hours, with the formation of a fibrinous exudate. Usually the symptoms, and any discomfort felt, passed away in two or three days. On the other hand, in realy healthy persons a reaction was distinctly rare.

Further use has shown several possible sources of error. Thus Smithies and Walker[3] point out that if the solution is not fresh, or is not homogeneous throughout, a patient may not get an effective dose. Failure may occur if it runs out or is wiped out of the eye, as may happen with a crying child. Again, the instillation should be done in the morning, to allow of the eye being watched during the rest of the day, or the reaction may be over before we see it.

In very cachectic patients, or those actually dying of phthisis, a reaction is rare, and, on the other hand, in some cases of syphilis or actinomycosis reactions may occur, just as tuberculin injections will at times react in the same class of patients. Other observers claim that reactions may be met with in typhoid, but this has been denied. Again, there is reason to suspect that when a markedly negative phase is present in phthisis, the reaction may be doubtful or delayed, though a definite result will follow if the test be repeated later on.

When these sources of error are avoided, have we a test which can be relied on ? The general opinion so far seems to be that it

[1] *Proc. Roy. Soc. Med.*, Path. Sect., 1907–08, i. 74.

[2] *Presse méd.*, June 17th, 1907. [3] *J. Am. M. Ass.*, 1908, l. 259.

is fairly accurate. The difficulty of verifying the results is indeed very great. For tuberculosis varies enormously. There may be a slight and quickly cured local lesion, or a widespread and fatal affection. Before a doubtful case which has been tested dies, a slight lesion may have entirely healed, or a new infection may have taken place. It is probable that a really cured case of phthisis does not give a reaction, although the marks left by the disease may be evident *post-mortem*. Thus at the Mount Vernon Hospital numbers of negative results were found in convalescents, though every one of the active cases with bacilli was positive.

On examining the statistics, it is lamentable to find that hundreds, and perhaps thousands, of cases are useless for comparison, because no common form of classification has been adopted. The same difficulty vitiated many of the early sanatorium statistics. Some writers use various strengths of tuberculin in succession, as Eisen did, without mentioning which was successful. Many do not distinguish between known and doubtful cases of tuberculosis which were tested. The confusion in even the best papers is inconceivable, and most writers are more interested in mentioning special cases than in giving the comparative results.

It has been claimed that in proved tuberculosis a positive reaction occurs in about 95 per cent. of the cases, and upon taking a large number of recent reports, which were more or less uniform in method, and after eliminating the doubtful reactions and separating where possible the doubtful from the proved tubercular cases, this claim was substantiated. Thus for 1,072 cases given by English, French and American writers, there were[1] :—

| | | | | |
|---|---|---|---|---|
| (A) | Definitely tuberculous | 472 | = | 455 positive, or 96 per cent. |
| (B) | Doubtfully tuberculous | 124 | = | 78 positive, or 63 per cent. |
| (C) | Non-tuberculous | 476 | = | 15 positive, or 3.3 per cent. |

Though it is impossible to be sure of the accuracy of the previous diagnosis in every case, these results are sufficiently striking.

After all, the great question is, What is the value of the test in the borderland cases of tuberculosis ? Is it one which will enable us to decide definitely when all the other means of diagnosis fail, and to recognise the earliest cases of disease ? The really important point is its accuracy in the mildest forms of infection. We do get some slight evidence on this subject. Paul Eisen[2] obtained his highest percentage of reactions in the very earliest cases of phthisis (Turban's Stage I.). The previous diagnosis, in default of physical signs, was here confirmed by tuberculin

1 Smithies and Walker, *loc. cit.* ; Webster and Kilpatrick, *Brit. M. J.*, 1907, ii. 1644 ; Austin and Gruiibaum, *Proc. Roy. Soc. Med.*, Path. Sect., 1907–08, i. 74 ; Maclennan, *Brit. M. J.*, 1907, ii. 1642 ; Letulle, *Presse méd.*, July 3rd, 1907 ; Meille, *Brit. M. J.*, 1908, i. epit. p. 5 ; Stephens, *ibid*, 1908, i. 742.

2 *Beitr. z. Klin. d. Tuberk.*, 1907, Hft. 4.

injections, and the positive results were 78 per cent., as against 70 in Stage II., and 50 in Stage III. It is clear that the most urgent need is for an accurate register of very early cases and their results, with subsequent tests by other methods, if the delicacy of the reaction in these liminal instances is to be relied upon. Dr. Edgeworth has recommended that the opsonic index of doubtful cases which give a positive reaction should be taken on several occasions afterwards. Great variations in the opsonic index will fully confirm the tuberculous nature of the disease. Where an instillation into the eye is objectionable, H. de C. Woodcock suggests an alternative. He produces on the chest two small blisters. To one of them, on the second day, he applies Koch's new tuberculin (T.R.), allows it to dry, and then covers both with sterilised lint. The next day the one so treated is red with a surrounding halo if tuberculosis is present.

G. PARKER.

